# Participation in a clinical trial of a text messaging intervention is associated with increased infant HIV testing: A parallel-cohort randomized controlled trial

**DOI:** 10.1371/journal.pone.0209854

**Published:** 2018-12-31

**Authors:** Thomas A. Odeny, Elizabeth A. Bukusi, Elvin H. Geng, James P. Hughes, King K. Holmes, R. Scott McClelland

**Affiliations:** 1 Department of Medicine, University of Missouri-Kansas City, Kansas City, MO, United States of America; 2 Center for Microbiology Research, Kenya Medical Research Institute, Nairobi, Kenya; 3 Department of Epidemiology, University of Washington, Seattle, WA, United States of America; 4 Department of Global Health, University of Washington, Seattle, WA, United States of America; 5 Department of Obstetrics and Gynecology, University of Washington, Seattle, WA, United States of America; 6 Division of HIV/AIDS, Department of Medicine, San Francisco General Hospital, San Francisco, CA, United States of America; 7 Department of Biostatistics, University of Washington, Seattle, WA, United States of America; 8 Department of Medicine, University of Washington, Seattle, WA, United States of America; 9 Center for AIDS Research, University of Washington, Seattle, WA, United States of America; 10 Institute of Tropical and Infectious Diseases, University of Nairobi, Nairobi, Kenya; The Ohio State University, UNITED STATES

## Abstract

**Objective:**

Text messages significantly improve uptake of infant HIV testing in clinical trial contexts. Women who were excluded from a randomized trial in Kenya were followed to create a comparison between women who were enrolled and did not receive the study SMS intervention and women who were screened but not enrolled.

**Design:**

Parallel-cohort randomized controlled trial analysis.

**Methods:**

We compared time to infant HIV testing between women in three groups: the Trial SMS group, the Trial Control group, and the Comparison Cohort comprised of women who were screened but not enrolled.

**Results:**

Of the 1,115 women screened, 388 (35%) were eligible for trial enrollment, and were randomized to receive either intervention text messages (Trial SMS; N = 195) or continue usual care (Trial Control; N = 193). Among 727 women not enrolled in the study (Comparison Cohort), we obtained infant HIV testing data from clinic records for 510 (70%). The cumulative probability of infant HIV testing was highest in the Trial SMS group (92.0%; 95% CI 87.5–95.3), followed by the Trial Control group (85.1%; 95% CI 79.5–89.8), and lowest among women in the Comparison Cohort (43.4%; 95% CI 39.2–47.8).

**Conclusions:**

Both the Trial SMS group and the Trial Control group were significantly more likely to have their infants tested for HIV compared to the Comparison Cohort, providing evidence of a “clinical trial effect.” This analysis suggests that SMS interventions should be implemented as an adjunct to consistent and engaged delivery of basic health services.

## Introduction

Early diagnosis of HIV infection and prompt initiation of antiretroviral therapy (ART) among infants born to women living with HIV reduces infant mortality by 76% [1]. Despite the efficacy of this intervention, effectiveness in practice is diminished due to low rates of early infant HIV testing. For example, in 2015 only 44% of HIV-exposed infants in Kenya were tested for HIV within the recommended two months of birth [2]. To address this problem, a randomized trial was conducted to evaluate short message service (SMS) text messages to improve uptake of infant HIV testing in Kenya [3, 4]. The study found that SMS text messaging significantly improved rates of infant HIV testing. Unexpectedly, the proportion of infants tested for HIV in the trial control group was higher than the proportions reported by programs offering routine PMTCT services in Kenya during the same period. In addition, a number of patients were screened and not enrolled into the study, including 53% who were excluded due to having a gestational age less than 28 weeks. We followed these excluded patients to create a comparison group, which could be assessed in relation to the Trial Control group as well as the Trial intervention (SMS) group. The hypothesis was that there would be a progressive, stepwise increase in infant HIV testing when comparing non-trial participants (reference category) to infants in the control and intervention groups of the trial. Differences in the outcome could offer an estimate of the magnitude of the “clinical trial effect.” In addition, this analysis may provide broader insights for a range of studies carried out in resource limited settings, where outcomes may be sensitive to inputs from clinical trial environments.

## Methods

### Design and participants

In this “parallel-cohort RCT” analysis [5], the primary exposure was participation in the randomized efficacy trial of the TextIT strategy (ClinicalTrials.gov NCT01433185). The design, analysis, and results of the trial are presented elsewhere [3, 4]. Briefly, HIV-infected pregnant women in prevention of mother-to-child HIV transmission (PMTCT) programs were randomly assigned to receive either theory-based two-way text messages during pregnancy and the postpartum period (intervention arm) or usual care (control arm). Eligible women were 18 years of age or older, pregnant at a gestational age of 28 weeks or greater (or had delivered on the day of enrollment), enrolled in the PMTCT program, planning to remain in the study area, and had access to a mobile phone plus reported ability to read or have someone who read text messages on their behalf. Women who reported sharing phones were ineligible unless they had disclosed their HIV status to the person sharing the phone. The intervention consisted of 14 text messages sent at weeks 28, 30, 32, 34, 36, 38, 39, and 40 during pregnancy, and weeks 1, 2, 3, 4, 5, and 6 after delivery. Messages were tailored by inserting the mother’s and infant’s name, the infant’s sex, and allowing participants to choose their preferred language and time for receiving messages. The primary trial outcome was the proportion of infants undergoing HIV virologic testing within 8 weeks after birth, assessed using an intention-to-treat analysis. Ethical review committees of the Kenya Medical Research Institute, the University of Washington, and the University of California San Francisco approved the trial. All participants provided written informed consent.

This novel “parallel-cohort RCT” design aims to maximize inclusion of the population at risk of disease to make information from the original TextIT trial more applicable during potential scale up of the intervention [5]. Women who were recruited and screened for the randomized trial but did not meet trial inclusion criteria were selected as the comparison group for this secondary analysis. The primary outcome was time to infant HIV testing, defined as obtaining a dried blood spot sample for HIV virologic testing within 8 weeks after birth.

### Study procedures

Baseline demographic and clinical characteristics for trial participants were collected using a questionnaire administered prior to randomization, while those for non-trial participants were extracted from trial screening logs, patient charts, antenatal care clinic registers, and electronic medical records. Follow-up and primary outcome information for both trial and non-trial participants were extracted from the HIV-exposed infants (HEI) register, HEI patient chart, health facility maternity register, postnatal clinic register, and PMTCT clinic patient charts. Data extraction was done approximately six months after analysis and publication of the initial randomized trial.

### Statistical analysis

Baseline characteristics were summarized using descriptive statistics, and differences across the three study groups were compared using chi-square tests. The three study groups included women receiving the SMS intervention (Trial SMS), women enrolled in the trial control group (Trial Control), and women screened but not enrolled (Trial Ineligible). Time to infant HIV testing in the three study groups was compared using the Kaplan-Meier method and log-rank tests. Hazard ratios and associated 95% confidence intervals (CI) were estimated using a Cox proportional hazards regression model. Time to infant HIV testing was measured in days from the date of birth to the date when a dried blood spot (DBS) sample was collected for HIV virologic testing. In routine practice, DBS samples are collected six weeks after birth to coincide with infant immunization clinic schedules. However, DBS collection before or after six weeks would still be valid for determining infant HIV status. Survival time was censored at eight weeks or at the time of maternal or infant death. The proportional hazards assumption was assessed using tests and graphs based on scaled Schoenfeld residuals [6].

Potential confounding factors were included in the final regression model using a sequential approach by first applying background knowledge using directed acyclic graphs (DAGs), and then using statistics-based methods to determine adjustment variables [7, 8]. In the first step, variables were selected for model inclusion based on *a priori* importance of being potential confounders of the association between trial participation and infant HIV testing [9]. These variables included trial eligibility criteria, age and education level of the mother, receiving ART (because of CD4 count <350 or stage 3 or 4 disease, which were indications for ART, independent of PMTCT, at the time of the trial [10]), year of maternal enrollment into PMTCT, maternal knowledge about PMTCT, and receiving ART prophylaxis for PMTCT (AZT during pregnancy, AZT+3TC+NVP at delivery, AZT+3TC after delivery, and infant NVP; as opposed to receiving ART because of CD4 count <350 or stage 3 or 4 disease). The relationships between these variables were summarized and analyzed qualitatively using a DAG (S1 Fig) [11]. Based on this DAG, age, education, and enrollment in the PMTCT program were determined to be sufficient for adjustment to control for confounding.

In the second multivariable model-building step, a Cox proportional hazards model with a backward stepwise model building approach was used. All three variables determined in the first step were included in this second step. Variables that changed the estimated hazard ratio of the main effect by ≥10% were retained [12]. In the end, both age and education were dropped from the model. The final model adjusted only for enrollment in the PMTCT program (versus pregnant women living with HIV who were not enrolled to receive PMTCT services at the time of screening).

To investigate whether results were biased by the high proportion of non-trial participants with missing outcome information, sensitivity analyses were performed that considered them all first as failures (no infant HIV testing by 8 weeks), and then as successes (infant HIV testing completed by 8 weeks). The multivariable regression models for these sensitivity analyses were unadjusted, given that non-trial participants without outcome information were also missing baseline demographic and clinical characteristics.

We report unadjusted and adjusted hazard ratios and corresponding 95% CIs. All tests were two-sided and a significance level of *P* < 0.05 was used. Stata v13 was used to perform all statistical analyses (StataCorp, College Station, TX).

## Results

### Study participants

**Fig 1:**
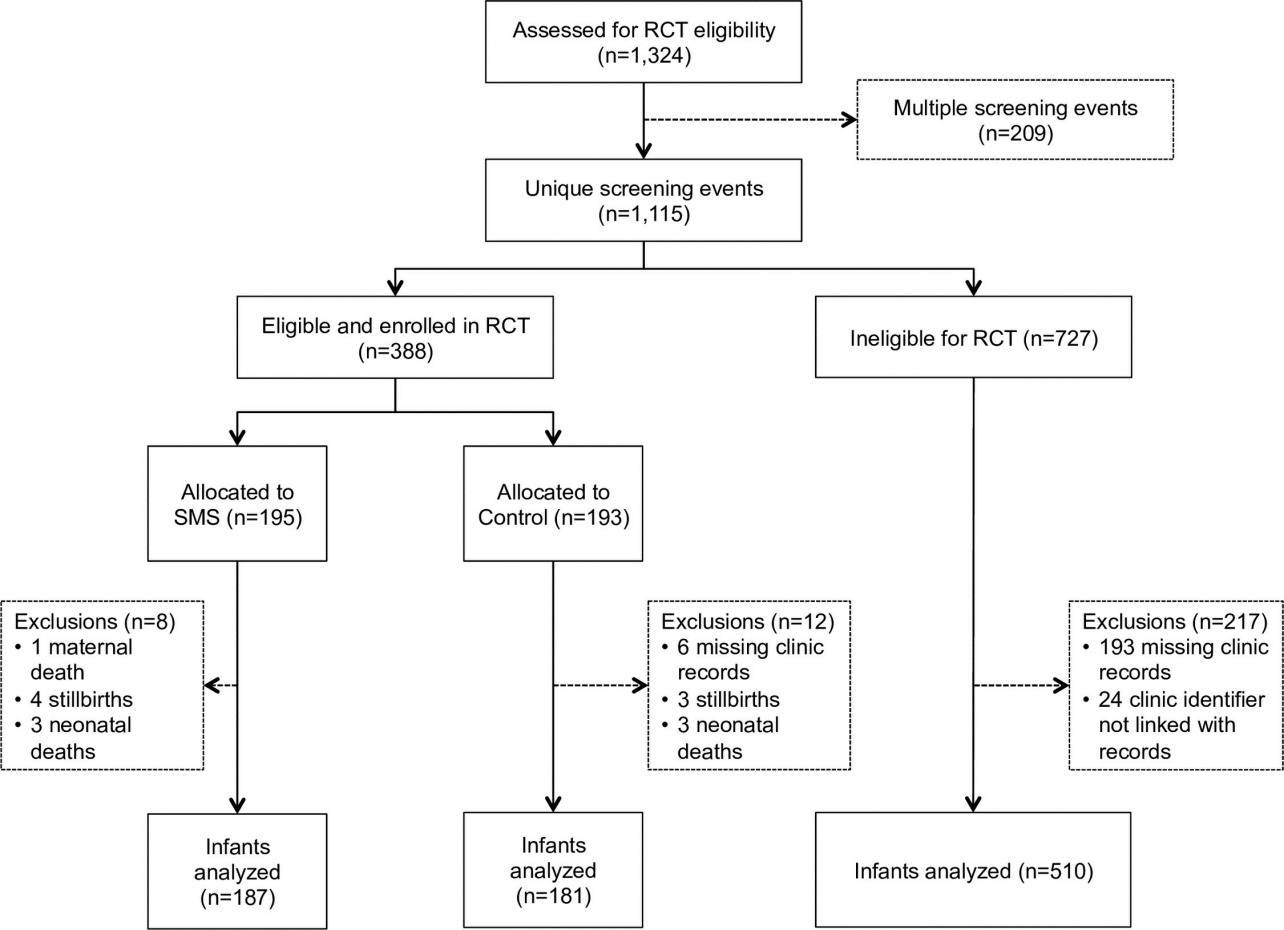
Study profile.

There were 1,324 screening visits by 1,115 HIV-positive pregnant women attending antenatal clinic between April and October 2012 (Fig 1). Of these, 388 women were eligible for trial enrollment, and all were randomized to receive the intervention text messages (Trial SMS; N = 195) or continue usual care (Trial Control; N = 193). The remaining 727 women were ineligible for trial enrollment for various reasons, including 550 (76%) with gestation <28 weeks and 170 (23%) who did not have access to a phone. Among those ineligible for the trial, 217 patient records could not be located. This precluded ascertainment of both their baseline and follow-up information. Trial eligibility criteria collected by study staff at screening did not differ significantly when comparing these 217 women without clinic records to other trial-ineligible women whose records were available (Table 1).

**Table 1:** Trial eligibility criteria among women screened but not enrolled comparing those with clinic records available versus those without records.

	Records Available (N = 510)	Records Not Available (N = 217)	Chi-square p-value
	N (%)	N (%)	
Adult 18 or older	503 (98.6)	211 (97.2)	0.20
Able to read SMS	505 (99)	217 (100)	0.14
Gestation ≥28 weeks	121 (23.7)	56 (25.8)	0.55
Enrolled in PMTCT program	497 (97.5)	207 (95.4)	0.15
Has access to mobile phone and if shared has disclosed HIV status	393 (77.1)	164 (75.6)	0.67
Willing to receive text messages	491 (96.3)	215 (99.1)	*0*.*04*
Planning to remain in study area during study	499 (97.8)	214 (98.6)	0.49

SMS = short message services; PMTCT = prevention of mother-to-child transmission of HIV

Baseline characteristics comparing trial participants to trial-ineligible women included in this analysis are shown in Table 2. Overall, the median maternal age was 26 years (interquartile range [IQR] 23–30), 110 (12%) were employed, 540 (60%) had primary or greater level of education, 760 (11%) were married, and 148 (16%) were primigravidae. The median CD4 cell count at screening was 459 cells/μL (IQR 320–620), 151 (17%) were classified as having WHO stage 3 or 4 disease, and 424 (47%) were receiving ART because of CD4 count <350 or stage 3 or 4 disease, which were indications for ART, independent of PMTCT, at the time of the trial [10].

**Table 2:** Baseline demographic and clinical characteristics comparing trial participants versus those ineligible for trial participation.

	Trial participants		Ineligible (N = 510)	Chi-square p-value
	Control (N = 193)	SMS (N = 195)		
	N (%)	N (%)	N (%)	
Maternal age (years)				
<18	0 (0)	0 (0)	9 (1.8)	0.08
18–24	65 (33.7)	60 (30.8)	188 (36.9)	
25–34	111 (57.5)	111 (56.9)	267 (52.4)	
35+	17 (8.8)	24 (12.3)	46 (9)	
Data missing	0	0	0	
Education				
None	3 (1.6)	3 (1.5)	18 (3.5)	<0.01
Primary	110 (57)	115 (59)	119 (23.3)	
Secondary	55 (28.5)	64 (32.8)	26 (5.1)	
Post-secondary	25 (13)	13 (6.7)	13 (2.5)	
Data missing	0	0	334 (65.5)	
WHO stage				
1	103 (53.4)	110 (56.4)	262 (51.4)	0.77
2	57 (29.5)	55 (28.2)	116 (22.7)	
3	27 (14)	23 (11.8)	74 (14.5)	
4	6 (3.1)	7 (3.6)	14 (2.7)	
Data missing	0	0	44 (8.6)	
Most recent CD4 cell count				
<200	18 (9.3)	22 (11.3)	46 (9)	0.89
200–349	38 (19.7)	40 (20.5)	80 (15.7)	
350–500	55 (28.5)	54 (27.7)	119 (23.3)	
500+	82 (42.5)	78 (40)	206 (40.4)	
Data missing	0	0	59 (11.6)	
Employed (vs. not employed)	39 (20.2)	35 (17.9)	36 (7.1)	0.64
Data missing	0	0	346 (67.8)	
Luo ethnicity (vs. other)	177 (91.7)	188 (96.4)	384 (75.3)	0.01
Data missing	0	0	115 (22.5)	
Married (vs. not married)	28 (14.5)	31 (15.9)	40 (7.8)	0.01
Data missing	0	0	39 (7.6)	
First pregnancy (vs. >1)	29 (15)	27 (13.8)	92 (18)	0.25
Data missing	0	0	15 (2.9)	
No previous deliveries (vs. ≥1)	29 (15)	27 (13.8)	93 (18.2)	0.22
Data missing	0	0	15 (2.9)	
Receiving ART (vs. no ART)	102 (52.8)	101 (51.8)	221 (43.3)	0.42
Data missing	1 (0.5)	0	123 (24.1)	
Received AZT prophylaxis	81 (42)	85 (43.6)	240 (47.1)	<0.01
Data missing	1 (0.5)	0	154 (30.2)	
Received AZT+3TC+NVP (delivery pack)	53 (27.5)	60 (30.8)	237 (46.5)	<0.01
Data missing	1 (0.5)	0	157 (30.8)	
Received AZT+3TC (post-delivery pack)	51 (26.4)	60 (30.8)	238 (46.7)	<0.01
Data missing	1 (0.5)	0	157 (30.8)	
Prophylaxis for baby issued	133 (68.9)	139 (71.3)	384 (75.3)	<0.01
Data missing	2 (1)	0	113 (22.2)	
HIV test done on screening day	5 (2.6)	5 (2.6)	87 (17.1)	<0.01
Data missing	0	0	58 (11.4)	
HIV counseling with partner	49 (25.4)	40 (20.5)	147 (28.8)	<0.01
Data missing	0	0	60 (11.8)	

ART = antiretroviral therapy

Trial-ineligible women had higher proportions of missing data across all baseline variables, compared to trial participants (S2 Fig). However, the distribution of maternal age, employment status, gravidity, parity, WHO clinical stage, most recent CD4 cell count, and reason for being on ART (low CD4/stage 3 or 4 disease versus PMTCT prophylaxis) did not differ significantly compared to trial participants (Table 2). There were higher proportions of women with primary or greater level of education in the trial SMS and control groups compared to the trial-ineligible group (98%, 99%, and 31% respectively; p<0.01). The proportion of married women was two times higher in the trial SMS and control groups compared to the trial-ineligible group (15%, 16%, and 8% respectively; p<0.01).

### Time to infant HIV testing

**Fig 2:**
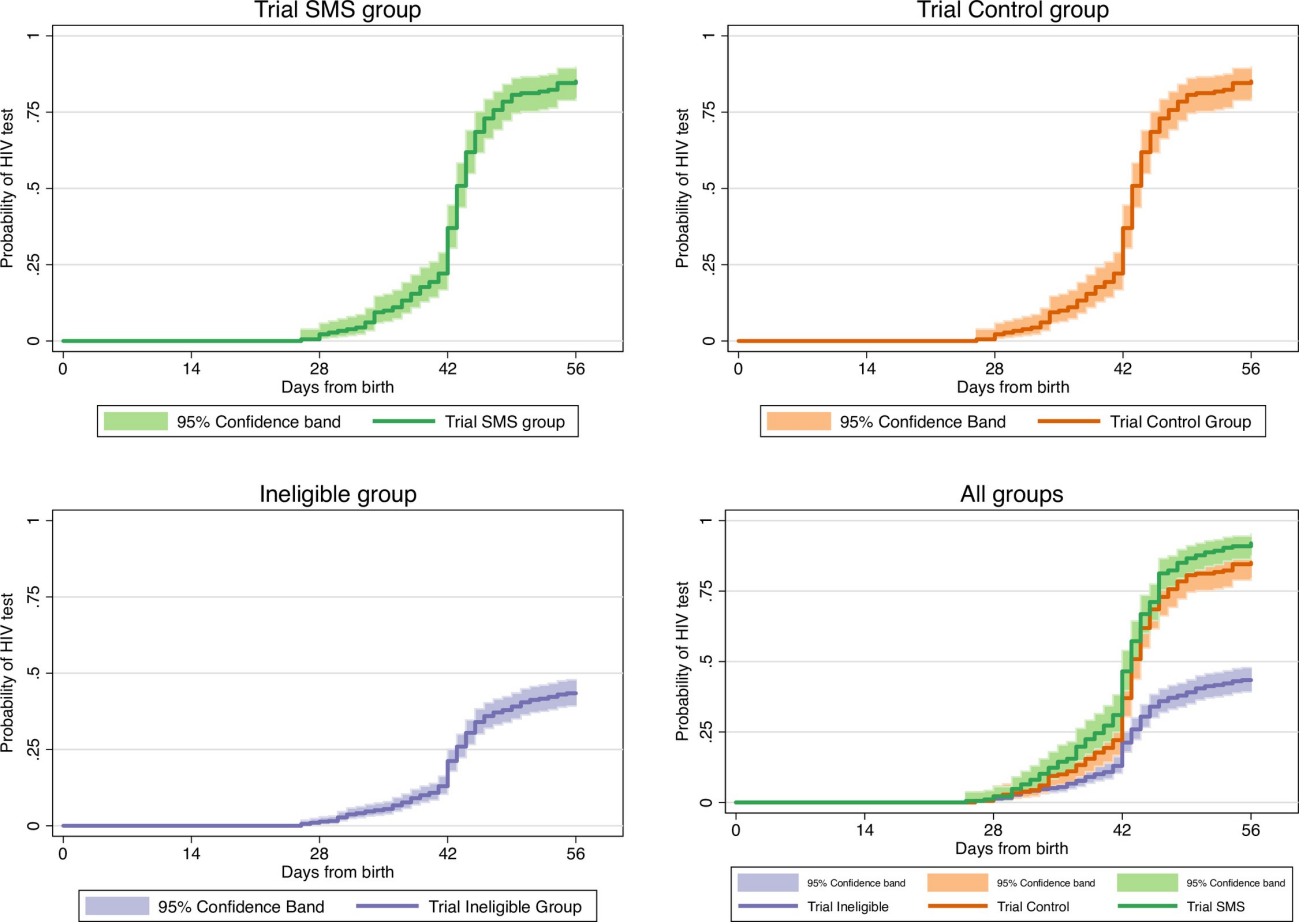
Kaplan-Meier plots showing time from birth to infant HIV virologic testing comparing trial participants in the SMS group, trial participants in the control group, and trial-ineligible participants.

Overall, the median time from birth to infant HIV testing was 46 days (interquartile range [IQR] 42–56). The Kaplan-Meier estimates of the cumulative probability of infant HIV testing were highest among trial participants in the SMS group (92.0%; 95% CI 87.5–95.3), followed by trial participants in the control group (85.1%; 95% CI 79.5–89.8), and lowest among trial-ineligible women (43.4%; 95% CI 39.2–47.8) (log-rank p-value<0.0001; Fig 2). There was a stepwise increase in the adjusted hazard ratios of infant HIV testing when comparing the trial-ineligible group (reference category) with the trial control group (hazard ratio [HR] 2.82; 95% CI 2.29–3.48), and with the trial SMS group (HR 3.48; 95% CI 2.84–4.27).

### Determinants of infant HIV testing

In Cox proportional hazards models (Table 3), there was a statistically significant stepwise increase in the rates of infant HIV testing with increasing maternal age. Compared to women younger than 18 years, there were higher rates of infant HIV testing in women aged 18–24 years (HR 3.38; 95% CI 0.84–13.64; p = 0.09), 25–34 years (HR 4.24 95% CI 1.06–17.04; p = 0.04), and older than 35 years (HR 5.77; 95% CI 1.41–23.56; p = 0.02). Similarly, higher education levels were associated with a stepwise increase in rates of infant HIV testing. Compared to women who reported no education, women who had primary education (HR 1.63; 95% CI 0.95–2.80; p = 0.07), secondary education (HR 1.69; 95% CI 0.97–2.96; p = 0.04), and post-secondary education (HR 2.16; 95% CI 1.19–3.94; p = 0.01) had higher rates of infant HIV testing. Women who were tested for HIV (i.e. received a new diagnosis of HIV infection) on the day of screening for trial enrollment were also significantly more likely to have their infants tested for HIV compared to those who had been tested previously (HR 2.06 95% CI 1.49–2.85; p<0.001). Women who had a record of having received ART for clinical indications prior to pregnancy were also significantly more likely to have their infants tested for HIV compared to those not receiving prior ART (HR 1.44; 95% CI 1.21–1.71).

**Table 3:** Determinants of time to infant HIV virologic testing in Cox proportional hazards regression models.

Variable	Unadjusted		Adjusted *	
	HR (95% CI)	p-value	HR (95% CI)	p-value
Exposure group *				
Ineligible	Ref.		Ref.	
Trial Control	2.85 (2.31–3.50)	*<0*.*001*	2.82 (2.29–3.48)	*<0*.*001*
Trial SMS	3.51 (2.87–4.30)	*<0*.*001*	3.48 (2.84–4.27)	*<0*.*001*
**Eligibility criteria**				
Enrolled in PMTCT program *	2.36 (0.88–6.32)	0.09	1.43 (0.53–3.84)	0.5
Adult 18 years or older	5.71 (0.80–40.62)	0.08		
Able to read SMS	1.87 (0.47–7.51)	0.38		
Gestation ≥28 weeks	2.29 (1.91–2.75)	*<0*.*001*		
Has access to mobile phone and if shared has disclosed HIV status	2.06 (1.54–2.80)	*<0*.*001*		
Willing to receive text messages	0.88 (0.51–1.53)	0.66		
Planning to remain in study area during study	0.78 (0.39–1.58)	0.50		
**Baseline socio-demographic and clinical characteristics**					
Maternal age (years)				
<18	Ref.			
18–24	3.38 (0.84–13.64)	*0*.*09*		
25–34	4.24 (1.06–17.04)	*0*.*04*		
35+	5.77 (1.41–23.56)	*0*.*02*		
Employed	1.11 (0.88–1.42)	0.38		
Education				
None	Ref.			
Primary	1.63 (0.95–2.80)	*0*.*07*		
Secondary	1.69 (0.97–2.96)	*0*.*06*		
Post-secondary	2.16 (1.19–3.94)	*0*.*01*		
Ethnicity (Luo vs. other)	1.51 (1.02–2.22	*0*.*04*		
Married (vs. not married)	0.80 (0.62–1.03)	0.08		
Second or higher pregnancy	1.10 (0.87–1.39)	0.82		
1+ previous deliveries	1.10 (0.87–1.38)	0.43		
WHO stage				
1	Ref.			
2	1.16 (0.95–1.41)	0.15		
3	1.26 (0.99–1.60)	0.06		
4	1.26 (0.80–1.98)	0.32		
Most recent CD4 cell count (cells/μL)			
<200	Ref.			
200–349	0.83 (0.60–1.14)	0.25		
350–500	0.90 (0.67–1.21)	0.49		
500+	0.86 (0.65–1.14)	0.31		
HIV test on the date of screening for trial (new HIV diagnosis)	2.06 (1.49–2.85)	*<0*.*001*		
HIV counseling with partner	1.19 (0.99–1.43)	*0*.*06*		
Receiving ART for own health	1.44 (1.21–1.71)	*<0*.*001*		
Received AZT prophylaxis during pregnancy	0.66 (0.56–0.79)	*<0*.*001*		
Received AZT+3TC+NVP (delivery pack)	0.59 (0.49–0.70)	*<0*.*001*		
Received AZT+3TC (post-delivery pack)	0.59 (0.49–0.70)	*<0*.*001*		
Nevirapine prophylaxis for baby issued	0.67 (0.54–0.83)	*<0*.*001*		

*The final multivariable model included exposure group and enrollment in PMTCT program. Both educational level and age were dropped from the multivariable model during the model building process, as neither variable meaningfully impacted the association between exposure group and HIV virologic testing of the infant.

### Sensitivity analysis

The Kaplan-Meier plots of cumulative probabilities of infant HIV testing demonstrating the range of sensitivity analysis are shown in Fig 2. When the 217 ineligible women whose clinic files could not be located were all considered as having infants who failed to receive HIV testing, the cumulative probability of infant HIV testing among all trial-ineligible women reduced from 43.4% to 30.4% (95% CI 27.2–33.9). The hazard ratios for infant HIV testing increased to 4.34 (95% CI 3.52–5.35; p<0.001) comparing trial-ineligible women with trial control participants, and to 5.34 (95% CI 4.35–6.56; p<0.001) comparing trial-ineligible women with trial SMS participants. In contrast, when the 217 trial-ineligible women without data were considered as all having received infant HIV testing, the cumulative probability of infant HIV testing among all trial ineligible women increased from 43.4% to 60.3% (95% CI 56.8–63.9). The hazard ratio for infant HIV testing comparing trial control participants with trial-ineligible women was lower, but remained statistically significant (HR 1.68; 95% CI 1.40–2.02; p<0.001), as did the hazard ratio comparing trial SMS participants with trial-ineligible women (HR 2.07; 95% CI 1.73–2.47; p<0.001).

## Discussion

In this study, we found that enrollment in a clinical trial, regardless of receiving the intervention or control condition, was associated with a significant increase in early infant HIV testing by eight weeks after delivery. Compared to trial-ineligible participants who were similar sociodemographically, women who enrolled in the trial but did not receive the study intervention were about three times more likely to have their infants tested for HIV. Irrespective of trial participation, higher maternal age and higher levels of education were both significantly associated with higher likelihood of infant HIV testing.

The large effect of trial participation implies that in frail health systems, where women face confusing and complicated experiences of health care, modest activities to engage them can have large effects. What did the trial offer to women that had such a profound effect on outcomes? Three relatively simple practices likely resulted in the observed effects: intensive one-on-one counseling during the informed consent process, greater care in data collection and record completion by regular program staff for women known to be in the trial, and changes in the behavior of participating women and non-study staff due to observation.

In this study, non-participants in the trial were relatively similar to participants, including a substantial proportion who were excluded due to gestational age. Therefore, observed differences are unlikely due to selection. It is more likely that differences in outcomes (the “trial effect”) were a result of the package of services women experienced through participation in the trial. This “trial effect” is the difference between the non-enrolled Comparison Cohort and the Trial Control group, whereas the intervention (SMS) effect is the difference between the Trial Control and the Trial SMS groups. The “trial effect” observed suggests that the role of SMS in retention interventions should be considered as a part of an approach where the basics are present. For example, a health facility able to offer SMS should have enough coordination, efficiency, and quality to make use of this technology. These findings reinforce the idea that interventions should be considered in the context of core services.

These results should be interpreted in the context of a number of limitations. First, outcomes for approximately 30% of trial-ineligible women who did not have clinic records available could not be ascertained. Excluding them might lead to selection bias. Importantly, sensitivity analyses were conducted across the widest possible range of scenarios, and found that interpretation of the main findings remained similar under both the most optimistic and most pessimistic assumptions. The problem of missing data in this analysis highlights one of the important challenges of using routinely collected data for research. The quality of data collected routinely for PMTCT and other HIV services is generally low compared to more rigorous data collection systems for research or surveillance [13–16]. Patient records may be incomplete, handwriting illegible, pages torn, or entire registers missing. These data problems may be due to a number of factors including competing clinical tasks, staff shortages, logistical difficulties in timely supply of data collection tools, and the fact that routinely collected data are rarely used to inform ongoing patient care [17]. A second limitation was that the comparison between women enrolled in the trial and those screened but not enrolled was not based upon randomization for purposes of comparison. Therefore, results could have been influenced by unmeasured confounding factors. To maximize the validity of results in the presence of residual confounding, carefully constructed multivariable models took into account known and potential confounding factors including trial eligibility criteria. An analysis that included eligible but non-enrolled women might have resulted in a better comparison group for trial participants. However, this was limited by full participation of eligible women at participating facilities and lack of data on eligibility among contemporaneous patients at non-participating facilities.

This study adds to a very limited literature in which investigators have compared outcomes between patients included and excluded from a randomized trial. Taken together with the findings of efficacy from the original randomized trial, the results of this analysis suggest that SMS interventions should essentially always be implemented as an adjunct to consistent and engaged delivery of basic health services. In this analysis, the sum of the “trial effect” and the “SMS effect” represents the potential gains that can be attained through a combination of quality basic services with SMS.

## Supporting information

S1 Fig Directed acyclic graph showing relationships between factors known or suspected to be associated with trial participation (exposure) and infant HIV testing (outcome). (TIF)

S2 Fig A “missingness map” showing patterns of missing data across baseline variables among trial-ineligible women. (TIF)
