# Supplementary figures and images for: Participation in a clinical trial of a text messaging intervention is associated with increased infant HIV testing: A parallel-cohort randomized controlled trial
Source: PLoS One. 2018 Dec 31;13(12):e0209854. doi: 10.1371/journal.pone.0209854 (PMC6312205; doi:10.1371/journal.pone.0209854)

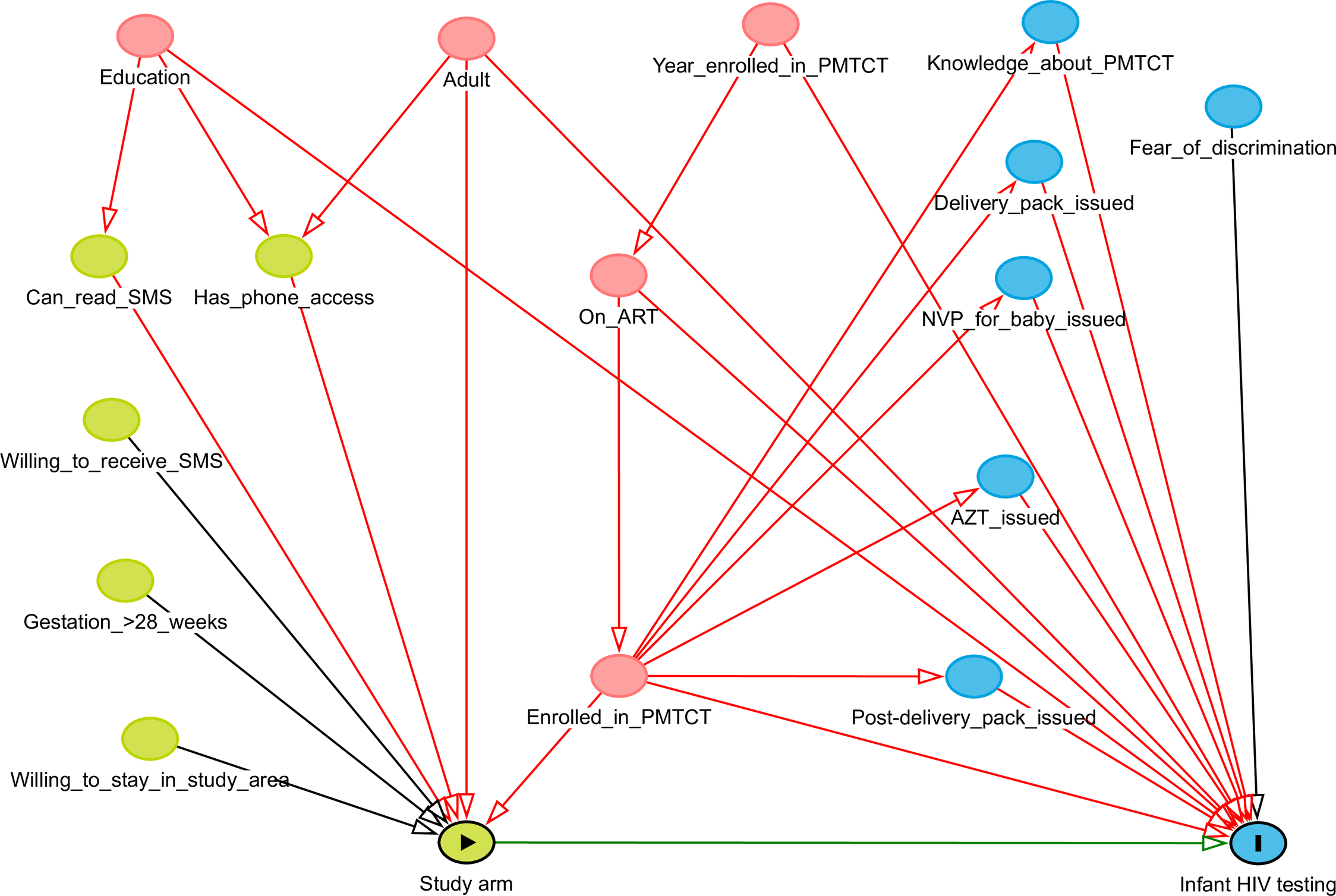

Supplement: S1 Fig — (TIF) [file pone.0209854.s001.tif]

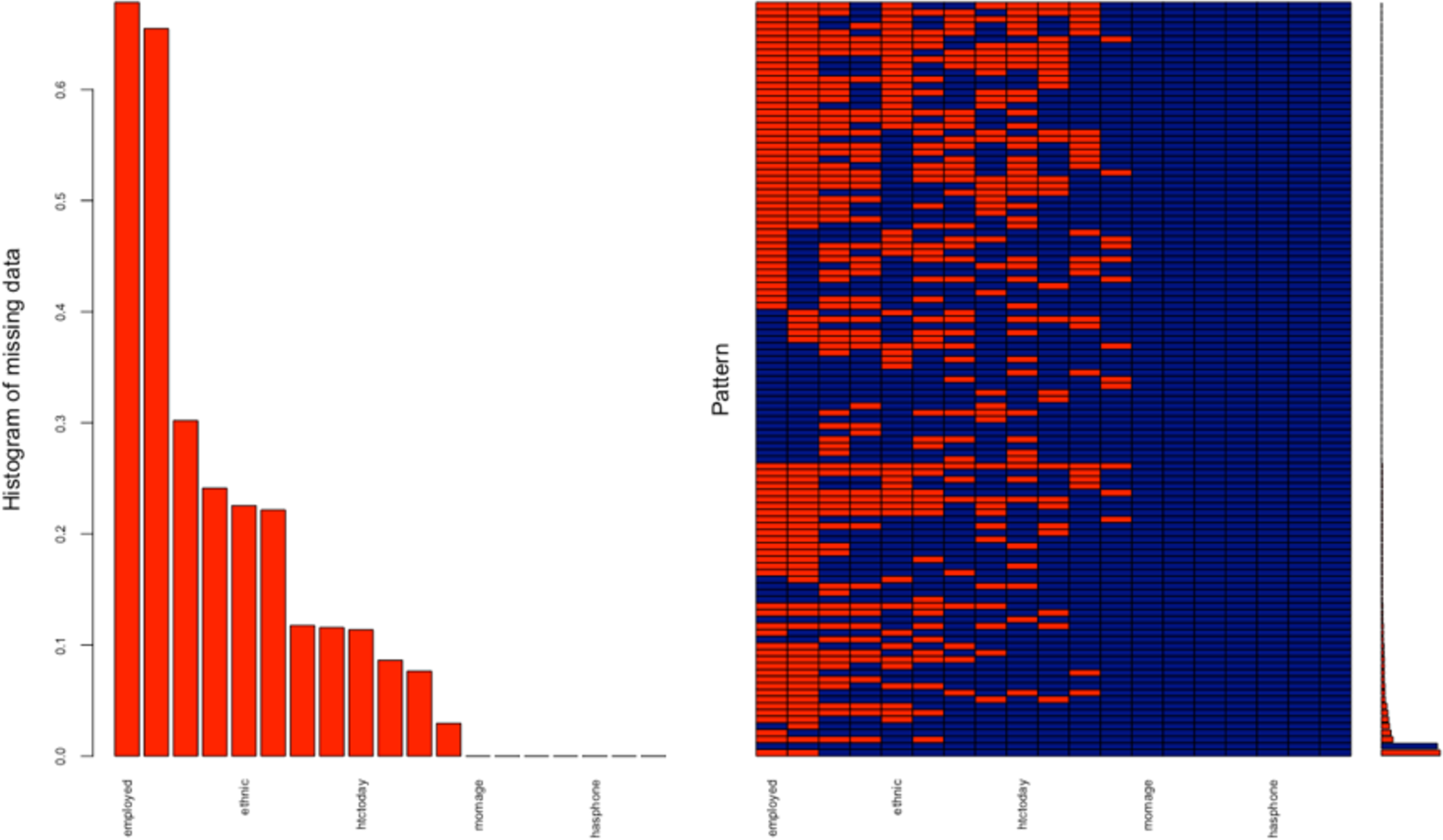

Supplement: S2 Fig — (TIF) [file pone.0209854.s002.tif]
